# Functional Polymorphism -31C/G in the Promoter of *BIRC5* Gene and Risk of Nasopharyngeal Carcinoma among Chinese

**DOI:** 10.1371/journal.pone.0016748

**Published:** 2011-02-03

**Authors:** Fuchao Ma, Hongxing Zhang, Yun Zhai, Wenfeng Huang, Chang Zhao, Shengqiu Ou, Hong Zhou, Wenzhao Yuan, Zhifu Wang, Hongxue Wang, Wei Yue, Lixia Yu, Peiyao Li, Xia Xia, Mi Cai, Yang Zhang, Ying Cui, Fuchu He, Yilong Ma, Gangqiao Zhou

**Affiliations:** 1 State Key Laboratory of Proteomics, Beijing Proteome Research Center, Beijing Institute of Radiation Medicine, Beijing, China; 2 Affiliated Cancer Hospital of Guangxi Medical University, Nanning, Guangxi, China; 3 Institutes of Biomedical Sciences, Fudan University, Shanghai, China; Universite de Montreal, Canada

## Abstract

**Background:**

Baculoviral inhibitor of apoptosis repeat-containing 5 (BIRC5, also called as survivin) is a member of the inhibitor of apoptosis protein (IAP) family, which plays an important role in the occurrence and progression of cancer. Recently, a polymorphism in the promoter of *BIRC5*, -31C/G (rs9904341), was shown to influence BIRC5 expression.

**Methods:**

We examined whether the -31C/G was related to the risk of developing nasopharyngeal carcinoma (NPC) in a case-control population from Guangxi province in southern China, which consists of 855 patients with NPC and 1036 controls. This polymorphism was genotyped by TaqMan assay. The genetic associations with the occurrence and progression of NPC were estimated by logistic regression.

**Results:**

We observed a statistically significant increased occurrence of NPC associated with the CC genotype (odds ratio [OR], 1.40; 95% confidence interval [CI], 1.13–1.73; P = 0.0020) compared with the genotypes containing G allele (CG + GG genotype). However, no significant association was observed for the -31C/G with the severity of NPC (as measured by tumor-node-metastasis staging system).

**Conclusion:**

Our findings suggest that the functional polymorphism -31C/G in the promoter of *BIRC5* gene may play a role in mediating the susceptibility to NPC among Chinese.

## Introduction

Nasopharyngeal carcinoma (NPC) is an epithelial malignancy rarely seen in most regions of the world but is particularly prevalent among populations from Southeast Asia. [1] Numerous environmental factors have been reported to confer the risk of developing NPC, including infection with the Epstein-Barr virus (EBV), long-term cigarette smoking, occupational exposure to formaldehyde, and various dietary factors. [2] Genetic factors may also contribute to the marked racial and geographic differences in NPC incidence, in that genetic polymorphisms of genes involving in different steps of carcinogenesis have been implicated in susceptibility to NPC. [3]–[14] The identification of susceptibility genes contributing to NPC would help to clarify pathogenesis of this malignancy and to predict individual and population risks of occurrence and progression of NPC.

It's well known that the apoptosis plays an important role in the development of cancers. [15] Baculoviral inhibitor of apoptosis repeat-containing 5 (BIRC5, also called survivin) is a novel member of the inhibitor of apoptosis protein (IAP) family, which is abundantly expressed in fetal tissues and in a variety of human malignancies, but almost undetectable in normal or well-differentiated adult tissues. [16] Overexpression of BIRC5 in various cellular systems has been found to be uniformly associated with inhibition of apoptosis, while the low expression of BIRC5 contributed to the enhanced spontaneous cell death. [17] Consistently, various studies of *Birc5* with conditional knockout mice have shown phenotypes with exaggerated apoptosis, with or without catastrophic mitotic defects. [18]–[21]

In a variety of human malignancies, the BIRC5 was frequently observed to be markedly over-expressed. [22] Furthermore, the BIRC5 expression has been positively correlated with poor prognosis of these cancers. [22] With regard to NPC, the elevated expressions of BIRC5 were also observed in tumor cells and tumor tissues compared with normal adjacent nasopharyngeal epithelium and tissues. [23], [24] For the outcome in NPC patients, the over-expression of BIRC5 was significantly associated with advanced clinical stage (III-IV), tumor stage (T3-4) and lymph node metastasis (N1-3), and lower 5-year survival rate. [23], [25] Furthermore, the expression of BIRC5 in NPC tissues was negatively correlated with the apoptosis index, which was equal to the total number of apoptotic cells or bodies over total number of tumor cells. [23], [25] Additionally, knock-down of *BIRC5* by RNA interference in NPC cell lines (C666-1) has shown decreased viability and increased apoptosis, suggesting a role for *BIRC5* in resistance to apoptosis in NPC. [24] The study in another NPC cell line (tet-on LMP1 HNE2) found that the latent membrane protein1 (LMP1) encoded by EBV could trigger the expression of BIRC5, which then promoted cell proliferation and inhibited apoptosis. [26] Taken together, these observations indicate that the BIRC5 plays an important role in the NPC occurrence and progression.

On the basis of above functional relevance of *BIRC5* both *in vitro* and *in vivo* in the pathogenesis of NPC, we hypothesize that the *BIRC5* may be an excellent biological candidate susceptibility gene for the NPC. It is expected that the functional polymorphisms within *BIRC5* could result in genotype-dependent difference in susceptibility to NPC.

Recently, a functional single nucleotide polymorphism (SNP), i.e. -31C/G (rs9904341), in the promoter of *BIRC5* gene was identified. [27] This polymorphism is located at the cell cycle-dependent elements (CDE) and cell cycle homology regions (CHR) repressor binding site of the promoter. Several studies have shown that the -31C/G polymorphism was associated with modified binding affinity of the CDE/CHR repressor, changed activity of the promoter, and subsequently resulted in different expression levels of *BIRC5* mRNA and protein in normal and cancer cells. [27]–[29] The -31C allele was shown to have a significantly higher transcriptional activity than the -31G allele, and carriers of the -31CC genotype had an increased BIRC5 levels than those of the GC and GG genotypes. [28]–[30] Therefore, it is hypothesized that this functional SNP in the *BIRC5* gene promoter may contribute to individual's tumor susceptibility. Indeed, several epidemiological studies have shown -31C/G to be associated with the risk of lung, [28] colorectal, [30] urothelial [31] and gastric cancer. [32] The role of −31C/G in NPC, however, has never been specifically investigated. In the present study, we examined whether the functional polymorphism in the *BIRC5* promoter, -31C/G, has any bearing on the occurrence or progression of NPC among Chinese.

## Materials and Methods

### Ethics statement

This study was performed with the approval of the Ethical Committee of Beijing Institute of Radiation Medicine (Beijing, China). At recruitment, written informed consent was obtained from all participants involved in this study.

### Study subjects

This case-control study included 855 incident patients with NPC and 1036 control subjects. Among them, a total of 477 patients with NPC and 480 controls were previously recruited between September 2003 and July 2005 at the Guangxi Cancer Hospital (Nanning, China). [3], [4] In this study, a further 378 patients with NPC were recruited from December 2005 to January 2008 at this hospital. All the 855 patients with NPC were newly diagnosed and pathologically confirmed, and were unrelated ethnic Chinese and residents in Nanning city and the surrounding regions. The response rate for the case patients was 95%. Patients that received chemotherapy or radiotherapy before surgery or had other type of cancer were excluded from the present study. Tumor staging was conducted according to the tumor-node-metastasis (TNM) classification by the 1997 American Joint Committee on Cancer (AJCC) system. [33] All TNM classifications were made by senior pathologists of the hospital based on the postoperative histopathologic examination. A further unrelated 556 cancer-free individuals were recruited as controls. All the 1036 controls were randomly selected from a community cancer screening program for early detection of cancer conducted in the same regions during the same time period as the NPC patients were collected. The selection criteria for the controls included no individual history of cancer and frequency matching to the cases on sex and age (±5 years). The response rate for control subjects was 92%.

At recruitment, personal information on demographic factors, medical history, and tobacco and alcohol use were collected via structured questionnaire. As described previously, individuals were considered smokers if they smoked up to 1 year before the date of cancer diagnosis for patients with NPC or up to the date of interview for controls. [3], [4] Information was collected on the number of cigarettes smoked per day, the age at which the individuals started smoking, and the age at which ex-smokers stopped smoking. Lighter or heavier smokers were categorized by the approximated 50th percentile pack-year value among controls [i.e., <24 or ≥24 pack-years; (cigarettes per day/20)× (years smoked)]. An alcohol drinker was defined as someone who consumed alcoholic beverages at least once per week for ≥6 months. The younger or older individuals were categorized by the approximated mean age value among controls [i.e., <45 or ≥45 years].

### Polymorphism genotyping

The -31C/G polymorphism (rs9904341) in the promoter of *BIRC5* gene was genotyped by TaqMan MGB technology (Assay ID: C_25474467_10; Applied Biosystems, Foster City, CA, USA) according to the manufacturer's protocol. Briefly, polymerase chain reaction (PCR) was performed in 5 µL volume with an initial 2 min at 50°C and 10 min at 95°C, followed by 40 cycles of 15 s at 95°C and 1 min at 60°C. The ABI PRISM 7900HT Fast Real-Time PCR System was used for the genotyping assay. Sequence Detection Systems software (SDS 2.3, Applied Biosystems) was used to automatically collect and analyze the data and to generate the genotype calls. Genotyping was performed in a blind manner so that the performers did not know the subjects' case or control status.

Case and control samples were distributed across each 384-well plate at random, with 20 randomly selected duplicate samples and 4 water controls per plate. The TaqMan assay of duplicate samples yielded a concordance rate of 100%. For further quality control, a 15% masked, random sample of cases and controls was tested by direct DNA sequencing and all results were 100% concordance. The PCR and sequencing primers were 5′-GTTCTTTGAAAGCAGTCGAG-3′ (Forward primer) and 5′-GCCAGTTCTTGAATGTAGAG-3′ (Reverse primer). PCR was performed with an initial denaturing step at 95°C for 3 min, 30 cycles of 94°C for 45 s, 57°C for 45 s, and 72°C for 1 min, and an extension step at 72°C for 10 min.

### Statistical analysis

Comparisons of sex, age, smoking and drinking status between patients and controls were performed using the *χ^2^* test. Differences of mean age and mean smoking level between patients and controls were analyzed by use of an unpaired *t* test. Genotype and allele frequencies for the -31C/G polymorphism were determined by gene counting. The significance of deviations from Hardy-Weinberg equilibrium was tested using the random-permutation procedure implemented in the Arlequin package (available at: http://lgb.unige.ch/arlequin/). The association between the genotypes and NPC risk was evaluated by multivariate logistic regression analyses. The odds ratios (OR) and 95% confidence intervals (CI) were adjusted for age, sex, smoking and drinking status, smoking level, nationality and family history where appropriate. Potential modification of the effect of -31C/G genotypes on the risk and progression of NPC was assessed for the above confounding factors by addition of interaction terms in the logistic model and by separate analyses of subgroups of individuals determined by these factors. A *P* value of <0.05 was used as the criterion of statistical significance, and all statistical tests were two sided. These analyses were performed using SPSS software (version 9.0; SPSS Inc., Chicago, IL).

## Results

The selected characteristics of patients with NPC and control subjects in Guangxi population are shown in Table S1. Overall, the controls were comparable with patients with regard to sex and tobacco and alcohol use. However, higher mean age was presented in the cases compared with controls (*P* = 0.0011, *t* test). A significantly higher proportion of the cases were of Non-Han nationality (*P* = 2.5×10^−14^) and positive for family history (*P* = 6.5×10^−5^) compared with controls. An absolute majority of the cases (97.0%) was classified as poorly differentiated squamous cell carcinoma. When stratified according to the TNM system, 4.8%, 46.2%, 30.3% and 18.7% of the patients had stage I, II, III and IV disease, respectively.

The genotyping results are shown in Table 1. This case-control study included 855 incident patients with NPC and 1036 control subjects. However, there were totally 26 samples that were unsuccessfully genotyped, and the percentages of these samples were similar in cases (1.3%) and controls (1.4%). Therefore, the actual sample size was 844 (98.7%) and 1021 (98.6%) for cases and controls, respectively. The observed genotype frequencies for the -31C/G polymorphism conformed to the Hardy-Weinberg equilibrium in both cases (*P* = 0.20) and controls (*P* = 0.85). The frequencies of the CC, CG, and GG genotype among cases were significantly different from those among controls (*χ^2^* = 9.064, *P* = 0.011, degree of freedom  = 2), and this difference was mainly caused by a higher frequency of the CC genotype among cases compared with controls (28.0% versus 21.9%). Based on logistic regression analysis with adjustment for age, sex, smoking and drinking status, smoking level, nationality and family history, the individuals carrying the -31CC genotype had a significantly increased susceptibility to NPC occurrence compared with those carrying the G allele (OR  = 1.40; 95% CI  = 1.13–1.73, *P* = 0.0020). When cases were limited to those with poorly differentiated squamous cell carcinoma (n = 829), the association results were similar (OR  = 1.40; 95% CI  = 1.13–1.74, *P* = 0.0020).

**Table 1:** Risk of NPC associated with the *BIRC5* -31C/G polymorphism in the Guangxi population.

Genotype	Cases, n (%)	Controls, n (%)
GG	205 (24.3)	273 (26.7)
CG	403 (47.7)	524 (51.3)
CC	236 (28.0)	224 (22.0)
OR (95% CI)		
CC vs. GG	1.41 (1.09–1.82)	
CC vs. CG	1.39 (1.11–1.75)	
CC vs. GG + CG	1.40 (1.13–1.73)	
*P* value		
CC vs. GG	0.0093	
CC vs. CG	0.0040	
CC vs. GG + CG	0.0020	

Due to genotyping failure, the actual sample size was 844 and 1021 for the cases and controls, respectively. OR, odds ratio; CI, confidence interval.

The ORs and *P* values were adjusted for age, sex, smoking and drinking status, smoking level, nationality and family history.

To further examine the associations between the -31C/G polymorphism and risk of NPC in the Guangxi population, we stratified the cases and controls by age, sex, smoking and drinking status, smoking level, nationality and family history (Table 2). Although the increased risk of NPC associated with the *BIRC5* -31CC genotype seemed to be more pronounced in subjects who were males, younger (<45 years), non-smokers, non-drinkers, Han Chinese, and negative for family history, these differences could be attributed to chance (all *P*≥0.05, test for homogeneity). Specially, we noted that although a significantly higher proportion of the cases were of Non-Han nationality compared to the controls (*P* = 2.5×10^−14^, Table 2), the frequencies of allele and genotype of the -31C/G polymorphism were comparable between Han and Non-Han groups, in both the cases and the controls (all *P*>0.05; Table S2). Therefore, the case and control populations were comparable in terms of their genetic ancestry in particular with respect to the -31C/G polymorphism. The slight difference in NPC risk between Han and Non-Han groups (*P*_homogeneity_  = 0.50, Table 2) further ruled out the influence of different ethnic ancestry on our results. Taking together, the *BIRC5* -31C/G polymorphism was an independent risk factor of NPC occurrence in the Guangxi population, with the potential confounding factors having no significant modification effect on it.

**Table 2:** Risk of NPC associated with the *BIRC5* -31C/G polymorphism by potential risk factors in the Guangxi population.

Category	CC^1^	CG + GG^1^	OR (95% CI)^2^	*P* value^2^	*P*_homogeneity_ 3
Sex					0.59
Male	168/153	443/583	1.45 (1.13–1.87)	0.0042	
Female	68/71	165/214	1.20 (0.81–1.79)	0.36	
Age					0.61
≥45	126/113	331/394	1.32 (0.98–1.77)	0.067	
<45	110/111	277/403	1.46 (1.07–1.98)	0.017	
Smoking status					0.19
Nonsmoker	166/146	424/566	1.56 (1.20–2.02)	0.0010	
Smoker	70/78	184/231	1.10 (0.75–1.62)	0.64	
Smoking level (pack-years)					0.72
≥24	30/27	75/96	1.48 (0.80–2.74)	0.21	
<24	40/51	109/135	0.92 (0.56–1.51)	0.73	
Drinking status					0.78
Nondrinker	160/155	432/566	1.37 (1.06–1.77)	0.015	
Drinker	76/69	176/231	1.43 (0.97–2.12)	0.075	
Nationality					0.50
Han	173/199	448/692	1.37 (1.08–1.74)	0.010	
Non-Han	63/25	160/105	1.68 (0.95–2.98)	0.077	
First-family history					0.49
Positive	16/9	38/20	1.66 (0.51–5.41)	0.40	
Negative	220/215	570/777	1.41 (1.13–1.75)	0.0022	

Due to genotyping failure, the actual sample size was 844 and 1021 for the cases and controls, respectively. OR, odds ratio; CI, confidence interval.

1 Number of genotype in cases/number of genotype in controls.

2 The ORs and *P* values were calculated by logistic regression with the CG + GG genotype as the reference group and adjusted for age, sex, smoking and drinking status, smoking level, nationality and family history where appropriate within the strata.

3 For difference in ORs within each stratum.

The population attributable fraction (PAF) associated with this genetic risk factor—the parameter that combines the strength of the epidemiological influence (relative risk) and the frequency of the genotype (population exposure rate)—can be estimated by the formula PAF  =  f(RR-1)/[1+f(RR-1)], where f is the population exposure rate, and RR is the relative risk (odds ratio). [34] The PAF calculated by the RR (OR, 1.40 [95% CI, 1.13–1.73]) (Table 1) combined with the frequency of the CC genotype (24.7%) (Table 1) indicates that 10.7% (95% CI, 3.9–18.0%) of the elevation in the risk of developing NPC can be attributed to the susceptibility effect of the -31CC genotype.

We also assessed the effect of the -31C/G genotype on severity of NPC (as measured by tumor-node-metastasis staging system) in the Guangxi population. However, the distributions of the -31C/G genotypes were not statistically significantly different among the subgroups with different clinical stage, or different T, N and M classification of the cancer (Table 3).

**Table 3:** Distributions of genotypes of the *BIRC5* -31C/G polymorphism among the subgroups with different severity of NPC in the Guangxi population

Stage of NPC	CC, n (%)	CG+GG, n (%)	*P* value	*χ^2^*	Degree of freedom
Clinical			0.946	0.372	3
I	12 (29.3)	29 (70.7)			
II	113 (28.8)	279 (71.2)			
III	68 (26.9)	185 (73.1)			
IV	43 (27.2)	115 (72.8)			
T			0.469	2.536	3
T1	45 (26.5)	125 (73.5)			
T2	121 (29.0)	296 (71.0)			
T3	42 (24.4)	130 (75.6)			
T4	28 (32.9)	57 (67.1)			
N			0.918	0.502	3
N0	50 (28.6)	125 (71.4)			
N1	116 (28.3)	294 (71.7)			
N2	47 (26.0)	134 (74.0)			
N3	23 (29.5)	55 (70.5)			
M			0.191	1.709	1
M0	233 (28.3)	591 (71.7)			
M1	3 (15.0)	17 (85.0)			

Due to genotyping failure, the actual sample size was 844 cases.

In 855 NPC patients of the Guangxi population, the mean ages at diagnosis (± SD, years) were 45.53 (±11.95) years for subjects with the CC genotype, and 46.78 (±11.94) years for those with the CG or GG genotypes. Therefore, subjects with the at-risk CC genotype were 1.25 years younger than those with the CG or GG genotypes by age at diagnosis, although the difference was not significant (*P* = 0.17, *t* test).

## Discussion

In this study, we assessed the associations of the functional polymorphism -31C/G in the promoter of *BIRC5* gene with the risk of occurrence and progression of NPC in the Guangxi population. No genetic association was found between this polymorphism and the progression of NPC. However, this polymorphism was significantly associated with the onset of NPC, with the individuals who carried the -31 CC genotype having an increased risk of occurrence of NPC, compared to those with the CG or GG genotypes. Subjects with the at-risk CC genotype tended to be younger than those with the CG or GG genotypes by age at diagnosis further supported a role of the *BIRC5* -31C/G polymorphism in the etiology of NPC. To our best knowledge, this is the first report of the genetic association between the *BIRC5* and NPC risk, confirming the initial hypothesis that the *BIRC5* may play a role in the pathogenesis of this malignancy.

The genetic association between *BIRC5* -31C/G and the onset of NPC is biologically plausible. BIRC5 is a member of IAP family and plays a key role in inhibiting apoptosis and promoting cell proliferation. [16], [35] The anti-apoptotic function of BIRC5 has been demonstrated both *in vitro* and *in vivo.* [17], [36] The mechanisms by which BIRC5 inhibits apoptosis are complicated, including regulation of the expression and function of BIRC5 to provide a different cell-survival threshold, physical interactions between BIRC5 and other molecules to exert anti-apoptotic effects, and compartmentalization of BIRC5 in mitochondria and release of the protein in the cytosol in response to the cell death stimuli. [22] In NPC, several studies have demonstrated that both mRNA and protein levels of BIRC5 were elevated in cancer tissues of NPC when compared with normal nasopharyngeal epithelium tissues. [23], [24] Furthermore, the expression of BIRC5 in NPC tissues was found to be negatively correlated with apoptosis index, [23], [25] and, the knockdown of *BIRC5* by RNA interference in NPC cell line showed decreased viability and increased apoptosis. [24]

Given the role of BIRC5 in the development of NPC, together with the earlier described functional relevance of -31C/G in modulation of BIRC5 expression, one might expect that individuals who carry the -31 CC genotypes, and thus have increased expression of BIRC5 and subsequently increased anti-apoptotic function, may be at a higher susceptibility to developing NPC. If carrying the -31 CC is regarded as a risk factor for the development of NPC, then the PAF calculated by the RR combined with the frequency of CC genotype indicates that 10.7% (95% CI, 3.9–18.0%) of elevation in the risk of developing NPC can be attributed to the susceptible effect of the -31CC genotype. At this stage, the *BIRC5* -31C/G polymorphism is unlikely to be appropriate for risk prediction testing. However, with more susceptibility loci being identified (e.g., by genome-wide association studies), and interaction effects among such loci together with other NPC risk factors taken into account, the prediction of NPC occurrence may become more accurate and clinically usable.

It has been reported that BIRC5 was markedly over-expressed in a broad range of human cancers. However, up to date, the *BIRC5* -31C/G polymorphism was only reported to be associated with risks of lung cancer, [28] urothelial carcinoma, [31] sporadic colorectal cancer, [30] gastric cancer [32] and NPC in the present study. The magnitude of NPC risk found with the CC genotype in the present study (OR  = 1.40, 95%CI: 1.13–1.73) was similar to the risk observed for lung cancer (OR  = 1.35, 95%CI: 1.04–1.75), but less than the risks observed for sporadic colorectal cancer (OR  = 2.68, 95%CI: 1.77–4.07), urothelial carcinoma (OR  = 4.0, 95%CI: 2.3–7.2), and gastric carcinoma (OR  = 4.83, 95%CI: 2.91–8.03) [28], [30]–[32]. However, given that the sample size of the later three studies were relatively small, the ORs in these studies might not indicate the real magnitudes of risk. Therefore, whether or not the magnitudes of risk reflect different roles of the -31C/G in different cancers warrants further investigation.

Furthermore, we have analyzed and compared the BIRC5 -31C/G polymorphism in populations from Taiwan (one of the regions with high NPC incidence), Hubei province (Central China) and Jiangsu province (Eastern China) based on available published data [31], [32], [37], and also compared the SNP with 11 populations from HapMap to see how different they are from our own study. As shown in Table S1, the allele and genotype frequencies of the *BIRC5* -31C/G polymorphism in our controls were similar to those of Mexicans and some populations of Chinese and African descent, but significantly different from those of other populations including Taiwan (*χ^2^* test). However, frequencies of the at-risk CC genotype were similar between controls from Taiwan and those in this study. It remains to be determined whether these differences between ethnic groups influence susceptibility to NPC.

Previous studies have reported that the *BIRC5* -31C/G polymorphism was associated with invasiveness or metastasis of some types of cancer. [31], [37] In NPC patients, the over-expression of BIRC5 has been shown to be extensively related to the poor outcome, including advanced clinical stage, tumor stage and lymph node metastasis, and lower 5-year survival. [23], [25] In the present study, however, we did not found any significant associations between the -31C/G polymorphism and the pathological stages of NPC. These findings may be due to the different molecular mechanisms of carcinogenesis among cancers and/or different ethnicities of study populations. In addition, these findings could be the result of the limited sample size and statistical power of our study. For example, this study has merely a power of 62.6% (I + II vs. III + IV), 56.1% (T1 + T2 vs. T3 + T4), 56.3% (N0 + N1 vs. N2 + N3) and 12.6% (M0 vs. M1) to detect an OR of 1.40 for carriers of the CC genotype relative to the carriers of the CG + GG genotypes. Therefore, we urge that the role of the -31C/G in the progression of NPC be investigated in additional studies with larger sample sizes.

In reviewing the results of this study, one must also keep two potential limits in mind. First, as a hospital-based study, our NPC cases were recruited from the hospital, while the controls were selected from the community population. Therefore, the inherent selection bias cannot be completely excluded. Well-designed and carefully executed multi-centre studies, with enrollment of large number of subjects, are warranted to draw conclusions which are more convincing and generalizable. Secondly, although the highly significant association between *BIRC5* and susceptibility to NPC is derived from a biologically based *a priori* hypothesis, our initial findings should be independently verified in other populations with a high incidence rate of NPC, such as other southern Chinese, Singaporeans, and Taiwanese. Therefore, any association reported in the present study should be interpreted with great caution, because without rigorous replication we cannot rule out the possibility that these findings are only due to chance.

In addition to BIRC5, many other candidate genes involving in different steps of carcinogenesis have also been implicated in NPC susceptibility. Further studies are needed to systematically verify these susceptibility genes, investigate the interactions between genetic and non-genetic risk factors, including dietary habit (e.g. salt fish) and EBV infection among others, and assess their contributions to the endemics of NPC among Chinese.

In conclusion, our results reveal an association for the first time between the functional polymorphism -31C/G in the promoter of *BIRC5* and susceptibility to NPC, and, the -31CC genotype, which are genetically predisposed to produce an increased expression level of BIRC5, seem to be a genetic risk factor for the NPC risk in a Chinese population. If confirmed by other studies, knowledge of genetic factors contributing to the pathogenesis of the NPC as presented here may have implications for the cancer screening and treatment of this disorder in the future.

## Supporting Information

Table S1 Of the 855 cases and 1036 controls involved in the present study, some were derived from our previous study [3], [4] and others were newly recruited. In cases, non-Han includes Zhuang (n = 211), Dong (n = 1), Hui (n = 1), Miao (n = 1), Mulao (n = 3) and Yao (n = 10) nationality; in controls, non-Han includes Zhuang (n = 132) nationality. Other histological types include vesicular nucleus cell carcinoma (n = 14), poorly differentiated adenocarcinoma (n = 4), and moderate differentiated squamous cell carcinoma (n = 5); and undifferentiated cancer (n = 3). Comparisons of sex, age, smoker, smoking level, and drinker distributions between patients and controls were performed by use of the *χ^2^* test. Differences of mean age and mean smoking level between patients and controls were analyzed by use of an unpaired *t* test. SD, standard deviation.
(DOC)

Table S2 The data of controls from Taiwan, Jiangsu and Hubei were derived from previously published studies [31], [32], [37]. ASW, African ancestry in southwest USA; YRI, Yoruba in Ibadan, Nigeria; LWK, Luhya in Webuye, Kenya; MKK, Maasai in Kinyawa, Kenya; CHB, Chinese Han in Beijing, China; CHD, Chinese in Metropolitan Denver, Colorado; GIH, Gujarati Indians in Houston, Texas; JPT, Japanese in Tokyo, Japan; CEU, Utah residents with northern and western European ancestry from the CEPH collection; TSI, Toscani in Italia; MEX, Mexican ancestry in Los Angeles, California.
(DOC)
