# Supplementary material for: Functional Polymorphism -31C/G in the Promoter of BIRC5 Gene and Risk of Nasopharyngeal Carcinoma among Chinese
Source: PLoS One. 2011 Feb 3;6(2):e16748. doi: 10.1371/journal.pone.0016748 (PMC3033414; doi:10.1371/journal.pone.0016748)
Supplement: Table S1 — Of the 855 cases and 1036 controls involved in the present study, some were derived from our previous study [3], [4] and others were newly recruited. In cases, non-Han includes Zhuang (n = 211), Dong (n = 1), Hui (n = 1), Miao (n = 1), Mulao (n = 3) and Yao (n = 10) nationality; in controls, non-Han includes Zhuang (n = 132) nationality. Other histological types include vesicular nucleus cell carcinoma (n = 14), poorly differentiated adenocarcinoma (n = 4), and moderate differentiated squamous cell carcinoma (n = 5); and undifferentiated cancer (n = 3). Comparisons of sex, age, smoker, smoking level, and drinker distributions between patients and controls were performed by use of the χ2 test. Differences of mean age and mean smoking level between patients and controls were analyzed by use of an unpaired t test. SD, standard deviation. (DOC) [file pone.0016748.s001.doc]

**Table S1.** Selected characteristics of patients with NPC and controls in the Guangxi population

| Category | Cases  (n = 855) | Controls  (n = 1036) | *P* value | *χ^2^* |
| --- | --- | --- | --- | --- |
| Sex, n (%) |  |  |  |  |
| Male | 616 (72.0) | 749 (72.3) | 0.90 | 0.015 |
| Female | 239 (28.0) | 287 (27.7) |  |  |
| Age, years |  |  |  |  |
| Mean (SD) | 46.5 (11.9) | 44.7 (12.1) | 0.0011 |  |
| ≥ 45, n (%) | 464 (54.3) | 516 (49.8) | 0.053 | 3.74 |
| < 45, n (%) | 391 (45.7) | 520 (50.2) |  |  |
| Smoking status, n (%) |  |  |  |  |
| Smoker | 257 (30.1) | 317 (30.6) | 0.80 | 0.065 |
| Nonsmoker | 598 (69.9) | 719 (69.4) |  |  |
| Smoking level, pack-years |  |  |  |  |
| Mean (SD) | 22.9 (12.9) | 24.3 (18.4) | 0.30 |  |
| ≥ 24, n (%) | 151 (58.8) | 189 (59.6) | 0.83 | 0.044 |
| < 24, n (%) | 106 (41.2) | 128 (40.4) |  |  |
| Drinking status, n (%) |  |  |  |  |
| Drinker | 256 (29.9) | 302 (29.2) | 0.71 | 0.14 |
| Nondrinker | 599 (70.1) | 734 (70.8) |  |  |
| Nationality, n (%) |  |  |  |  |
| Han | 628 (73.5) | 904 (87.3) | 2.5 × 10^-14^ | 58.07 |
| Non-Han | 227 (26.5) | 132 (12.7) |  |  |
| First-family history, n (%) |  |  |  |  |
| Negative | 797 (93.2) | 1006 (97.1) | 6.5 × 10^-5^ | 15.96 |
| Positive | 58 (6.8) | 30 (2.9) |  |  |
| Histological type, n (%) |  |  |  |  |
| Poorly differentiated squamous cell carcinoma | 829 (97.0) |  |  |  |
| Others | 26 (3.0) |  |  |  |
| Clinical stage, n (%) |  |  |  |  |
| I | 41 (4.8) |  |  |  |
| II | 395 (46.2) |  |  |  |
| III | 259 (30.3) |  |  |  |
| IV | 160 (18.7) |  |  |  |
| Local tumor invasion (T classification), n (%) |  |  |  |  |
| T1 | 170 (19.9) |  |  |  |
| T2 | 424 (49.6) |  |  |  |
| T3 | 174 (20.4) |  |  |  |
| T4 | 87 (10.1) |  |  |  |
| Lymph node involvement (N classification), n (%) |  |  |  |  |
| N0 | 178 (20.8) |  |  |  |
| N1 | 414 (48.4) |  |  |  |
| N2 | 185 (21.6) |  |  |  |
| N3 | 78 (9.2) |  |  |  |
| Distance metastasis (M classification), n (%) |  |  |  |  |
| M0 | 835 (97.7) |  |  |  |
| M1 | 20 (2.3) |  |  |  |
