# Supplementary material for: Functional Polymorphism -31C/G in the Promoter of BIRC5 Gene and Risk of Nasopharyngeal Carcinoma among Chinese
Source: PLoS One. 2011 Feb 3;6(2):e16748. doi: 10.1371/journal.pone.0016748 (PMC3033414; doi:10.1371/journal.pone.0016748)
Supplement: Table S2 — The data of controls from Taiwan, Jiangsu and Hubei were derived from previously published studies [31], [32], [37]. ASW, African ancestry in southwest USA; YRI, Yoruba in Ibadan, Nigeria; LWK, Luhya in Webuye, Kenya; MKK, Maasai in Kinyawa, Kenya; CHB, Chinese Han in Beijing, China; CHD, Chinese in Metropolitan Denver, Colorado; GIH, Gujarati Indians in Houston, Texas; JPT, Japanese in Tokyo, Japan; CEU, Utah residents with northern and western European ancestry from the CEPH collection; TSI, Toscani in Italia; MEX, Mexican ancestry in Los Angeles, California. (DOC) [file pone.0016748.s002.doc]

**Table S2.** The allele and genotype frequencies of the *BIRC5* -31C/G polymorphism in different populations.

| Populations | Sample size |  | Allele, n (%) | |  |  | Genotype, n (%) |  |
| --- | --- | --- | --- | --- | --- | --- | --- | --- |
|  |  |  | G | C |  | GG | CG | CC |
| In the present study |  |  |  |  |  |  |  |  |
| Controls | 1021 |  | 1070 (52.4) | 972 (47.6) |  | 273 (26.7) | 524 (51.3) | 224 (22.0) |
| Han | 891 |  | 931 (52.2) | 851 (47.8) |  | 239 (26.8) | 453 (50.8) | 199 (22.3) |
| Non-Han | 130 |  | 139 (53.5) | 121 (46.5) |  | 34 (26.2) | 71 (54.6) | 25 (19.2) |
| Cases | 844 |  | 813 (48.2) | 875 (51.8) |  | 205 (24.3) | 403 (47.7) | 236 (28.0) |
| Han | 621 |  | 596 (48.0) | 646 (52.0) |  | 148 (23.8) | 300 (48.3) | 173 (27.9) |
| Non-Han | 223 |  | 217 (48.7) | 229 (51.3) |  | 57 (25.6) | 103 (46.2) | 63 (28.3) |
| Previously published studies |  |  |  |  |  |  |  |  |
| Controls from Taiwan | 210 |  | 246 (58.6) | 174 (41.4) |  | 80 (38.1) | 86 (41.0) | 44 (20.9) |
| Controls from Jiangsu | 220 |  | 216 (49.1) | 224 (50.9) |  | 47 (21.4) | 122 (55.5) | 51 (23.2) |
| Controls from Hubei | 67 |  | 90 (67.2) | 44 (32.8) |  | 31 (46.3) | 28 (41.8) | 8 (11.9) |
| In HapMap |  |  |  |  |  |  |  |  |
| ASW | 53 |  | 57 (53.8) | 49 (46.2) |  | 12 (22.6) | 33 (62.3) | 8 (15.1) |
| YRI | 113 |  | 136 (60.2) | 90 (39.8) |  | 40 (35.4) | 56 (49.6) | 17 (15.0) |
| LWK | 90 |  | 105 (58.3) | 75 (41.7) |  | 32 (35.6) | 41 (45.6) | 17 (18.9) |
| MKK | 0 |  | 0 (0) | 0 (0) |  | 0 (0) | 0 (0) | 0 (0) |
| CHB | 84 |  | 83 (49.4) | 85 (50.6) |  | 21 (25.0) | 41 (48.8) | 22 (26.2) |
| CHD | 84 |  | 82 (48.8) | 86 (51.2) |  | 21 (25.0) | 40 (47.6) | 23 (27.4) |
| GIH | 85 |  | 121 (71.2) | 49 (28.8) |  | 44 (51.8) | 33 (38.8) | 8 (9.4) |
| JPT | 86 |  | 71 (41.3) | 101 (58.7) |  | 12 (14.0) | 47 (54.7) | 27 (31.4) |
| CEU | 113 |  | 162 (71.7) | 64 (28.3) |  | 60 (53.1) | 42 (37.2) | 11 (9.7) |
| TSI | 87 |  | 129 (74.1) | 45 (25.9) |  | 44 (50.6) | 41 (47.1) | 2 (2.3) |
| MEX | 48 |  | 54 (56.2) | 42 (43.8) |  | 16 (33.3) | 22 (45.8) | 10 (20.8) |
